# Co- and multimorbidity patterns in primary care based on episodes of care: results from the German CONTENT project

**DOI:** 10.1186/1472-6963-8-14

**Published:** 2008-01-18

**Authors:** Gunter Laux, Thomas Kuehlein, Thomas Rosemann, Joachim Szecsenyi

**Affiliations:** 1 Department of General Practice and Health Services Research, University of Heidelberg, Germany

## Abstract

**Background:**

Due to technological progress and improvements in medical care and health policy the average age of patients in primary care is continuously growing. In equal measure, an increasing proportion of mostly elderly primary care patients presents with multiple coexisting medical conditions. To properly assess the current situation of co- and multimorbidity, valid scientific data based on an appropriate data structure are indispensable. CONTENT (CONTinuous morbidity registration Epidemiologic NeTwork) is an ambitious project in Germany to establish a system for adequate record keeping and analysis in primary care based on episodes of care. An episode is defined as health problem from its first presentation by a patient to a doctor until the completion of the last encounter for it. The study aims to describe co- and multimorbidity as well as health care utilization based on episodes of care for the study population of the first participating general practices.

**Methods:**

The analyses were based on a total of 39,699 patients in a yearly contact group (YCG) out of 17 general practices in Germany for which data entry based on episodes of care using the International Classification of Primary Care (ICPC) was performed between 1.1.2006 and 31.12.2006. In order to model the relationship between the explanatory variables (age, gender, number of chronic conditions) and the response variables of interest (number of different prescriptions, number of referrals, number of encounters) that were applied to measure health care utilization, we used multiple linear regression.

**Results:**

In comparison to gender, patients' age had a manifestly stronger impact on the number of different prescriptions, the number of referrals and number of encounters. In comparison to age (β = 0.043, p < 0.0001), multimorbidity measured by the number of patients' chronic conditions (β = 0.51, p < 0.0001) had a manifestly stronger impact the number of encounters for the observation period. Moreover, we could observe that the number of patients' chronic conditions had a significant impact on the number of different prescriptions (β = 0.226, p < 0.0001) as well as on the number of referrals (β = 0.3, p < 0.0001).

**Conclusion:**

Documentation in primary care on the basis of episodes of care facilitates an insight to concurrently existing health problems and related medical procedures. Therefore, the resulting data provide a basis to obtain co- and multimorbidity patterns and corresponding health care utilization issues in order to understand the particular complex needs caused by multimorbidity.

## Background

Based upon technological progress and improvements in medical care and health policy, a growing number of patients survive medical conditions that used to be fatal formerly. As a result of this, an increasing proportion of mostly elderly primary care patients presents with multiple coexisting medical conditions. A large number of epidemiological studies from several countries support this estimate [1-4]. It could be shown that the risk of avoidable admissions and preventable complications increases dramatically with the number of chronic conditions [4]. Generally, it becomes more and more important to understand the particular complex needs caused by multimorbidity. However, to properly assess the current situation of multimorbidity and to draw recommendations for improvement, valid scientific data are indispensable.

In the German health care system, the GP (general practitioner) has some kind of gate-keeper role since patients who visit a specialist, without visiting the GP in advance have to pay an additional fee. In consequence, most visits to specialists are preceded by a GP consultation. The medical insurances cover all costs of routine care, including all visits to GPs as well as to specialists. Only some complementary and alternative medical treatments are not covered. A medical un-insurance does not exist, since a recently enacted law forces everyone to insure himself. In Germany everyone has free and unlimited access to the medical system and the vast majority of people uses the GP as entry into this system.

Electronic patient records in Germany are predominantly used for billing purposes. Thus, hitherto existing German routine data are unlikely to yield a realistic and differentiated picture of morbidity and health care utilization in primary care.

CONTENT (CONTinuous morbidity registration Epidemiologic NeTwork) is an ambitious project in Germany to establish a system for adequate record keeping and analysis in primary care. A scientific network was established consisting of participating surgeries, scientists, and statisticians. The aims are strictly scientific and the underlying hypothesis is that the knowledge-gaining process can be accelerated by combining the experience of many, especially with respect to complex interactions of factors and the analysis of rare events. The CONTENT EPR [5] (Electronic Patient Record) is based on the *ICPC-2-R *[6] (International Classification of Primary Care, 2^nd ^Revision) and allows a documentation in an *episode of care *structure over time. ICPC was accepted by the World Health Organization (WHO) as a related classification to be used for health information recording in primary care. The electronic version of the 2nd ICPC edition (ICPC-2-E) is available for the use in electronic medical records [7]. Generally, there is a broad consensus that ICPC exactly meets the needs in primary care both in research as well as in practice and will add knowledge about morbidity patterns in this field.

An episode of care is defined as health problem from its first presentation by a patient to a doctor until the completion of the last encounter for it or presumably death, if the focal problem still exists [6]. An episode of care (in this study) actually includes all GP encounter elements – none from specialists. Medical documentation in an episode of care character facilitates an insight to concurrently existing health problems and related medical procedures (e.g. prescriptions, referrals and hospitalization) and therefore provides a basis to obtain multimorbidity patterns. The CONTENT database has already yielded analyses that were impossible to achieve from German routine health care data [8].

We used the term multimorbidity to describe the co-occurrence of two or more *chronic *conditions as defined by van den Akker et al. [1]. The term comorbidity is used to describe the co-occurrence of medical conditions additional to an index disease as defined by Feinstein [9].

This study aims to describe co- and multimorbidity as well as health care utilization based on episodes of care, taking into account age and gender for the study population of the first 17 participating general practices.

## Methods

A software module was developed to enable the coding of reasons for encounter, diagnoses and medical procedures with ICPC and assigning these issues to episodes of care. The module was integrated in an existing practice software to be used by voluntarily participating GPs. The extended practice software features a special function for data export based on XML (eXtensible Markup Language). The resulting data files are sent to the center in Heidelberg via email or upload to a dedicated server. In addition to the actual patient data the files contain meta data with information about the observation period and the surgery. To assure data quality the practices obtain feedback reports at regular intervals containing operating figures about episode based data entry with ICPC (e.g. the percentage of encounters without a documented reason for encounter). Moreover, these figures are discussed in periodic meetings with the GPs in order to continuously improve the data quality.

As a basic principle, only anonymized data are transmitted. For each patient, the CONTENT EPR contains a case number, the year of birth and the gender but not patients' names or addresses. Thus, it is not possible to determine a patient's identity and the implementation of extensive data security mechanisms is not needed. Moreover, the German Data Protection Act allows the transmission of anonymized patient data for scientific purposes without an explicit compliance of the patients. The study protocol was approved by the ethics committee of the University of Heidelberg (approval number 442/2005).

The data stem from a total of 39,699 patients in a Yearly Contact Group (YCG) out of 17 general practices with a total of 24 GPs located in 4 different federal states in West Germany with a concentration in Baden-Württemberg and Hessen. The YCG can be considered as an appropriate denominator since it is a good approximation of the "attending patients" in health systems with a patient list system [10,11].

ICPC and episode based data entry was performed between 1.1.2006 and 31.12.2006. For these patients data about age, gender and episode based diagnoses were available as well as the corresponding medical procedures (different prescriptions and referrals). The number of different prescriptions per patient was determined at the 4^th ^level of the ATC (Anatomical Therapeutic Chemical Classification). The 4^th ^level determines the chemical or therapeutic or pharmacological subgroup. This is the level usually used to count "number of different drugs" as it is the level which aggregates drugs just above their descriptive chemical substance. The underlying referral list included all referrals to specialists (incl. repeat referrals) for the observed YCG.

On the basis of ICPC codes for the presented sample it was possible to define chronic conditions by using the concept of O'Halloran et al. that regards diagnoses as well as few chronic symptoms and complaints [12].

In order to model the relationship between the explanatory variables (age, gender, number of chronic conditions) and the response variables of interest (number of different prescriptions, number of referrals, number of encounters) we used multiple linear regression. Odds ratios were calculated by logistic regression. On account of the cluster sample study design the calculations were adjusted for the cluster (i.e. practice) on the basis of calculated ICCs (IntraCluster-Correlations). Statistical calculations were performed with SPSS version 14.0 and SAS version 8.0.

The study protocol was approved by the ethics committee of the University of Heidelberg (approval number 442/2005).

## Results

For 39,699 patients in the YCG a total of 76,428 different episodes of care with an average of 1.87 ± 0.02 chronic conditions per patient were processed. 40.4% of the patients were male and 59.6% were female. The average age of the patients was 48.8 ± 0.17 years.

**Figure 1:**
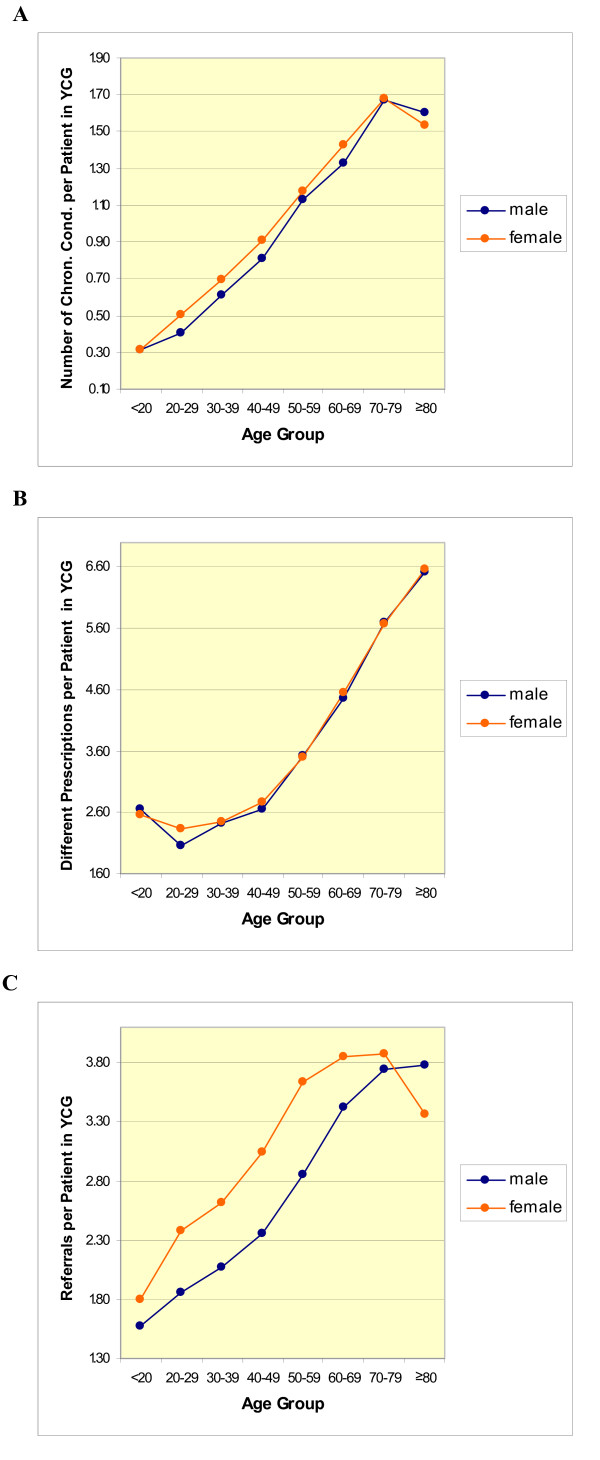
**Characteristics of multimorbidity and health care utilization**. A – Average number of different chronic conditions per patient in YCG (yearly contact group). B – Average number of different prescriptions per patient in YCG. C – Average number of referrals per patient in YCG.

To get an impression of multimorbidity regarding age and gender we considered the average number of different chronic conditions per patient in a YCG. Moreover, we considered the number of different prescriptions as well as the number of referrals per patient stratified for age and gender (Table 1 and Figures 1A to 1C). The figures already point in the direction that patient's age appears to play an important role in context with the above mentioned variables for both male and female patients.

**Table 1:** Characteristics of multimorbidity and health care utilization

**Age Group (Years)**	**Gender**	**Patients (%)**	**Average number of chronic conditions per patient in YCG (± SE)**	**Average number of different prescriptions per patient in YCG (± SE)**	**Average number of referrals per patient in YCG (± SE)**
**<20**	male	2316 (41.1)	0.32 ± 0.19	2.64 ± 0.21	1.57 ± 0.11
	female	3324 (58.9)	0.32 ± 0.18	2.56 ± 0.12	1.80 ± 0.12
**20–29**	male	1847 (37.9)	0.41 ± 0.21	2.05 ± 0.27	1.85 ± 0.13
	female	3028 (62.1)	0.50 ± 0.19	2.33 ± 0.15	2.38 ± 0.11
**30–39**	male	2110 (38.9)	0.61 ± 0.21	2.43 ± 0.23	2.07 ± 0.14
	female	3322 (61.1)	0.69 ± 0.18	2.45 ± 0.12	2.62 ± 0.09
**40–49**	male	2868 (41.5)	0.81 ± 0.17	2.64 ± 0.19	2.35 ± 0.12
	female	4051 (58.5)	0.91 ± 0.15	2.77 ± 0.13	3.04 ± 0.05
**50–59**	male	2324 (44.3)	1.13 ± 0.18	3.52 ± 0.19	2.85 ± 0.11
	female	2919 (55.7)	1.18 ± 0.17	3.50 ± 0.15	3.64 ± 0.12
**60–69**	male	2279 (44.9)	1.33 ± 0.19	4.45 ± 0.19	3.43 ± 0.11
	female	2791 (55.1)	1.43 ± 0.16	4.55 ± 0.16	3.85 ± 0.09
**70–79**	male	1568 (42.0)	1.67 ± 0.22	5.70 ± 0.31	3.75 ± 0.18
	female	2161 (58.0)	1.68 ± 0.19	5.67 ± 0.22	3.88 ± 0.12
**≥80**	male	737 (25.3)	1.60 ± 0.43	6.51 ± 0.69	3.78 ± 0.40
	female	2174 (74.7)	1.53 ± 0.21	6.57 ± 0.24	3.36 ± 0.15

To examine more precisely the dependence of the response variables (number of different prescriptions, number of referrals, number of encounters) on age, gender and number of chronic conditions we performed a multiple linear regression. Table 2 shows the corresponding results.

**Table 2:** Multiple linear regression analysis

**Response variable**	**Explanatory variables**	**Proportion of explained variance (adjusted R**^2)^	**Adjusted regression coefficient β**	**p**
**Different prescriptions**	Age	0.28	0.315	<0.0001
	Gender		0.008	n.s.
	Number of chronic conditions		0.226	<0.0001
**Referrals**	Age	0.39	0.178	<0.0001
	Gender		0.053	<0.0001
	Number of chronic conditions		0.300	<0.0001
**Encounters**	Age	0.37	0.043	<0.0001
	Gender		0.029	<0.0001
	Number of chronic conditions		0.510	<0.0001

Generally, in comparison to gender, the influence of age on the response variables was notably stronger, displayed by the regression coefficient β, that was standardized for the particular range. It could be observed that the impact of gender on the number of referrals (β = 0.053, p < 0.0001) was predominantly associated with referrals of women to gynaecologists.

In comparison to age (β = 0.043, p < 0.0001), multimorbidity had a manifestly stronger impact on the number of encounters (β = 0.51, p < 0.0001) within our regression model. Moreover, we could observe that the number of patients' chronic conditions had a significant impact on the number of different prescriptions (β = 0.226, p < 0.0001) as well as on the number of referrals (β = 0.3, p < 0.0001).

The CONTENT database also facilitates to describe the co-occurrence of medical conditions additional to an index disease. We wanted to describe the prevalence and the extent of comorbidities for the following highly prevalent chronic diseases: hypertension (K86/87), chronic ischemic heart disease (K74-76), diabetes mellitus (T89/90), and osteoarthrosis (L89-91). It has to be regarded that the prevalence estimates are based on the entry of the selected problem within the YCG in the observation period and do not refer to patients' lifetime. Table 3 shows that these chronic diseases feature a strong cohesion both for male and female patients. Moreover, the highly prevalent diseases lipid disorder (T93) and back syndrome (L84) were associated with these diseases.

**Table 3:** Prevalence and comorbidity based on ICPC

**Disease **(N = 39,699 patient years)	**n total**	**n males (% male)**	**n females (% female)**	**total/1000**	**males/1000**	**fe males/1000**	**Most frequent comorbidities male (%, odds ratio)**	**Most frequent comorbidities female (%, odds ratio)**
**K86/87 Hypertension**	2385	1033 (43.3)	1352 (56.7)	60.01	64.41	57.14	T93 Lipid Disorder 201(19.5, 8.4) K74-76 Chron.isc.heart d. 187(18.1, 10.9) T89/90 Diabetes mellitus 171(16.6, 7.7) L84 Back syndrome 140(13.6, 1.3) T99 Endocrine, other 79(7.6, 12.2) L89-L91 Osteoarthrosis 75(7.3, 3.6)	T93 Lipid Disorder 224(16.6, 8.1) L89-L91 Osteoarthrosis 222(16.4, 7.8) T89/90 Diabetes mellitus 221(16.3, 10.2) L84 Back syndrome 217(16.1, 1.7) K74-76 Chron.isc.heart d. 176(13.0, 14.0) P76 Depressive Disorder 119(8.8, 3.5)

**K74-76 Chronic ischemic heart disease**	955	507 (53.1)	448 (46.9)	24.06	31.61	19.93	K86/K87 Hypertension 159(31.4, 8.1) T93 Lipid Disorder 121(23.9, 9.5) T89/90 Diabetes mellitus 93(18.3, 7.5) L84 Back syndrome 92(18.1, 1.9) L89-L91 Osteoarthrosis 65(12.8, 6.8) K80 Cardiac arrhythmia 49(9.7, 9.9)	K86/K87 Hypertension 171(38.1, 12.5) L89-L91 Osteoarthrosis 97(21.7, 9.3) T89/90 Diabetes mellitus 90(20.1, 10.4) L84 Back syndrome 87(19.4, 2.0) T93 Lipid Disorder 81(18.1, 7.4) K77 Ischemic heart d. 71(15.8, 23.4)

**T89/90 Diabetes mellitus**	1225	566 (45.1)	659 (54.9)	31.61	35.29	27.85	K86/K87 Hypertension 152(26.9, 6.4) T93 Lipid Disorder 101(17.8, 6.3) K74-76 Chron.isc.heart d. 99(17.5, 8.3) L84 Back syndrome 70(12.4, 1.2) L89-L91 Osteoarthrosis 61(10.8, 5.5) T99 Endocrine, other 41(7.2, 8.6)	K86/K87 Hypertension 207(31.4, 9.5) T93 Lipid Disorder 99(15.0, 6.0) L89-L91 Osteoarthrosis 98(14.9, 5.8) K74-76 Chron.isc.heart d. 90(13.6, 11.2) L84 Back syndrome 88(13.4, 1.3) U99 Urinary disease, other 71(10.8, 2.7)

**L89-91 Osteoarthrosis**	1223	449 (36.7)	774 (63.3)	30.81	28.0	32.71	L84 Back syndrome 101(22.5, 2.6) K74-76 Chron.isc.heart d. 71(15.8, 6.9) K86/K87 Hypertension 69(15.3, 2.9) T93 Lipid Disorder 65(14.5, 4.6) T89/90 Diabetes mellitus 54(12.0, 4.2) Y85 Benign prost. hyp. 50(11.1, 7.2)	L84 Back syndrome 207(26.7, 3.2) K86/K87 Hypertension 202(26.1, 7.2) T93 Lipid Disorder 111(14.3, 5.8) K74-76 Chron.isc.heart d. 93(12.0, 9.7) T89/90 Diabetes mellitus 90(11.6, 5.4) U99 Urinary disease, other 84(10.8, 2.7)

## Discussion

As an answer to our research question we found a strong correlation between age, gender, multimorbidity and health care utilization. These findings are not surprising and do not stand in contrast to comparable findings in the international scientific literature [1-4,13]. Nonetheless, our study is the first approach to this phenomenon in our country with an international classification developed for primary care.

Generally, when addressing multimorbidity issues in order to compare the results of different studies, possible differences concerning the research question, the data sources and the definition of multimorbidity have to be taken into account. Fortin et al. collected prevalence estimations of multimorbidity in Europe, the Middle East, the United States, and Canada. Since research questions, information collection, and multimorbidity measures differed, major differences in the results were observed [14]. However, there is a broad consensus that multimorbidity and its high prevalence is an important issue in family practice that deserves more scientific research [15].

Van den Aker et al. concluded that multimorbidity, although it increases with age, is a frequent phenomenon among all ages [1]. This phenomenon was also observed within our study sample. 12.8% of the patients younger than 50 years featured 2 or more chronic conditions. Therefore, research into multimorbidity should not only focus on the elderly, who are especially at risk.

Multimorbidity as defined by routinely collected data in electronic patient records not only offers an epidemiological overview of morbidity patterns for the scientist, but can also help the GP to identify patients with an increased likelihood of needing more attention [16]. We observed a typical clustering of specific health problems (e.g. diabetes, hypercholesterinemia and hypertension, Table 3). These clusters can be easily identified by the GP on the basis of the EPR in order to apply an appropriate medical care and to initiate specific interventions (e.g. lifestyle modifications).

### Limitations

Generally, a potential selection bias must be admitted since the GPs' participation is voluntary and by now mainly focuses on Southwest Germany. Moreover, the number of 17 practices is still too small to draw strong conclusions.

In order to assess morbidity, there are several detailed and validated morbidity indexes [17]. For example, the "Cumulative Illness Rating Scale" (CIRS) [18] index additionally regards the severity of each condition and was also validated for the use to quantify multimorbidity for primary care patients [19]. However, since we had no information of the condition severity within the CONTENT EPR, we could not calculate this index for our study and had to limit on disease counts. Moreover, it would have been challenging to analyse the influence of sociodemografic factors (e.g. education, profession, income) on multimorbidity. However, sociodemografic information was only available for a small fraction of the sample.

The definition of a specific chronic condition on the basis of ICPC codes is often ambiguous. For example, we defined Osteoarthrosis (OA) by inclusion of ICPC codes L89 (Osteoarthrosis of hip), L90 (Osteoarthrosis of knee) and L91 (Osteoarthrosis, other). L84 (Back syndrome without radiating pain) is not included in our selection but also includes OA of the back. However, L84 also includes diseases that are not related to OA (e.g. back strain). Moreover, L91 includes 'arthritis unspecified' and 'traumatic arthropathy' that are not directly related to OA. This general problem could be solved by using a more specific terminology level which would allow grouping of all osteoarthrosis (no matter the site) from all applicable ICPC-2 codes.

### Strengths

As mentioned above, the CONTENT project is the first approach in Germany based on episodes of care and ICPC that facilitates detailed long term analyses of co- and multimorbidity.

Especially, the continuous registration of patients' presented symptoms is new in comparison to hitherto existing German EPRs. Thus, CONTENT data enable to analyse the correlation between presented symptoms and resulting diagnoses in consideration of existing comorbidities. Moreover, age, gender as well as seasonal and regional differences have to be taken into account. In the long run, for every ICPC symptom (*SY*) it will be possible to determine a list *L *of resulting diagnoses *D*_1_,....., *D*_*n *_and corresponding probabilities *P*_1_,....., *P*_*n *_taking into account the above mentioned constraints (A: age, G: gender, S: season, R: region, *C*_1_,....., *C*_*m*_: existing comorbidities), as the following formal description shows:

$$\begin{array}{cc} L(SY;A,G,S,R,\sum_{i=1}^{m}C_{i})=\{\begin{array}{ll} D_{1}: & P_{1}\\ D_{2}: & P_{2}\\ . & \\ . & \\ . & \\ D_{n}: & P_{n} \end{array}, & whereas\sum_{j=1}^{n}P_{j}=1 \end{array}$$

This detailed model represents an extension of the model presented by Lamberts et al. [20].

## Conclusion

We could observe a strong correlation between age, gender, multimorbidity and health care utilization in our study sample. Generally, documentation in primary care on the basis of episodes of care facilitates an insight to concurrently existing health problems and related medical procedures. Therefore, the resulting data provide a basis to obtain multimorbidity patterns and corresponding health care utilization issues. The continuously growing number of patients and practices has the potential to facilitate detailed long term analyses of co- and multimorbidity.

The increasing application of ICPC- and episode-based EPRs all over the world will allow challenging international comparisons in order to see national differences and regional distinctions and to discover what is generic in family practice and independent from local or national conditions. Further analyses will subsequently be based on the continuously expanding database and have the potential to shed light on complex epidemiological and health economics research questions.

## Competing interests

The author(s) declare that they have no competing interests.

## Authors' contributions

GL conceived and designed the study, organized and led data collection, analyzed and interpreted the data, and drafted the manuscript. TK assisted in data interpretation. TR assisted in study design and critically reviewed the manuscript. JS conceived and potentiated the superordinated project CONTENT. All authors read and approved the final manuscript.

## Pre-publication history

The pre-publication history for this paper can be accessed here:


